# A case of mammary analogue secretory carcinoma of the parotid gland: A case report

**DOI:** 10.1097/MD.0000000000020218

**Published:** 2020-07-10

**Authors:** Seong Hwan Kim, Min Eui Hong, Dong Jin Lee

**Affiliations:** a Department of Otolaryngology-Head and Neck surgery; b Department of Plastic and Reconstructive Surgery; c Department of Pathology, Kangnam Sacred Heart Hospital, Hallym University College of Medicine, Seoul, Korea.

**Keywords:** mammary analogue secretory carcinoma, parotid gland

## Abstract

**Rationale:**

Mammary analogue secretory carcinoma is a low grade salivary gland malignancy, first described by Skalova et al in 2010. The histological, immunohistochemical, and molecular characteristics of this tumor resemble those of secretory carcinoma of the breast.

**Patient concerns:**

A 40-year-old male patient without any specific past history came with complaints of 4 cm-sized hard, fixed, right infra-auricular mass without tenderness. There was no enlarged or enhancing lymph node in both neck.

**Diagnoses:**

Fine needle aspiration biopsy was done for right parotid mass and pathologic report was ”lymphocytes only” that suggested benign or low-grade malignant parotid tumor.

**Interventions:**

Partial parotidectomy including mass excision was performed. Operative finding showed hard bluish mass located in deep lobe of right parotid gland.

**Outcomes:**

Final pathologic report revealed secretory carcinoma of parotid gland. Homogenous eosinophilic secretions were identified inside microcystic structure. The immunophenotype was positive for epithelial membrane antigen, vimentin, S-100 protein. After 15 months of the surgery, the patient showed negative evidence of disease state.

**Lessons:**

We present here a case of mammary analogue secretory carcinoma of the parotid gland to help further characterize this rare tumor.

## 1 Introduction

Mammary analogue secretory carcinoma (MASC) is a newly recognized tumor of the salivary gland, characterized by morphologic and immunohistochemical features that strongly resembles a secretory carcinoma of the breast.^[1]^ In the past, most of MASC mainly have been diagnosed as acinic cell carcinomas because of some histologic overlap between these two tumors. However, MASC have an ETV6-NTRK3 gene fusion resulting from the t(12;15)(p13;q25) chromosomal translocation.^[1]^ This fusion gene encodes a chimeric tyrosine kinase that is known to play an important role on its oncogenesis.^[2]^ Immunohistochemical similarities between MASC and secretory carcinoma of the breast also include being S100 protein, epithelial membrane anti-gen (EMA), and vimentin positive and ”triple negative” (ER/PR/Her2negative).^[3]^ MASC predominantly affects men and normally does not behave in an aggressive way.^[4]^ The parotid gland is the most common affected gland by MASC.^[5]^ We present here a case of MASC occurring in a 40-year-old, male patient that manifested an asymptomatic mass in the right infra-auricular region and discuss cytological diagnostic clues for differential diagnosis between secretory carcinoma and other malignant tumors of the parotid gland.

## 2 Methods

**Figure 1:**
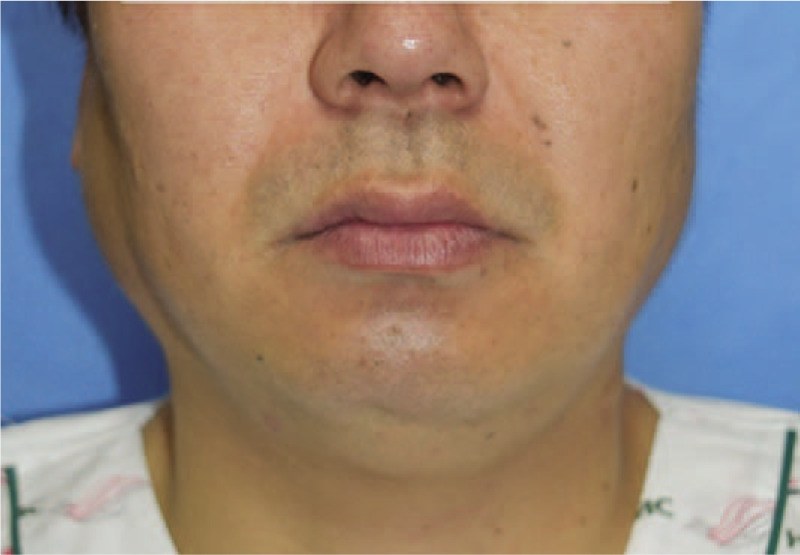
Photograph of patient with 4.0 cm sized right infra-auricular mass.

**Figure 2:**
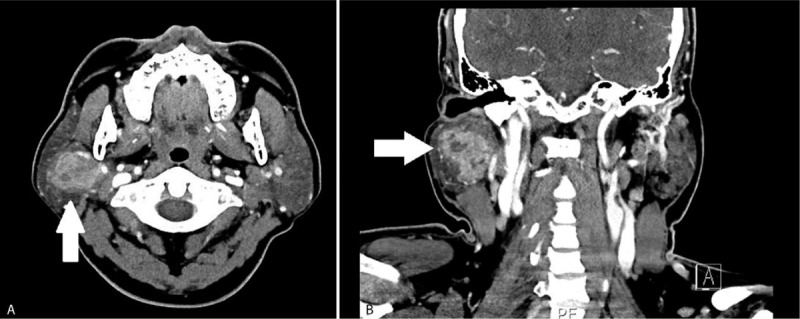
A and 2B. Neck CT revealed about 4.0 cm sized irregular marginated heterogenous enhancing mass in right parotid gland (the white arrow).

**Figure 3:**
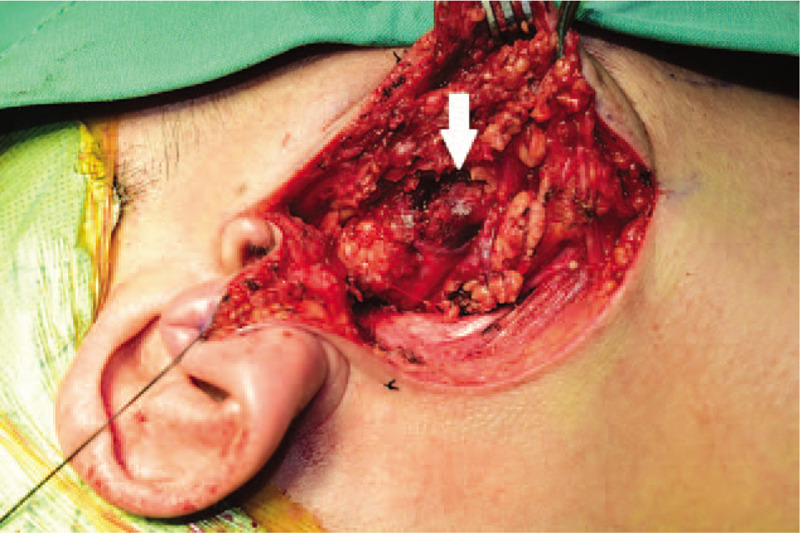
Operative finding showed hard bluish mass located in deep lobe of right parotid gland. There was severe adhesion between mass and facial nerve (the white arrow).

**Figure 4:**
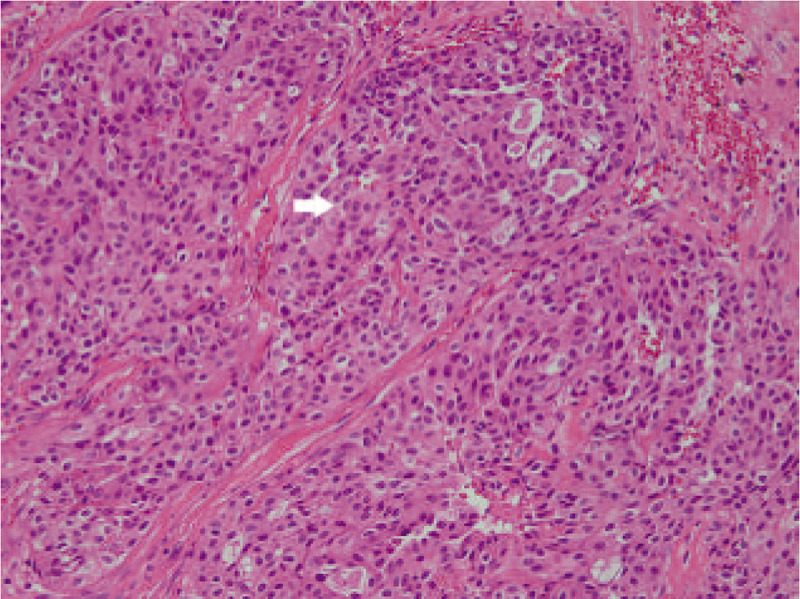
Hematoxylin and eosin staining. The neoplastic cells have round nucleus, with predominant eosinophilic cytoplasm (the white arrow).

**Figure 5:**
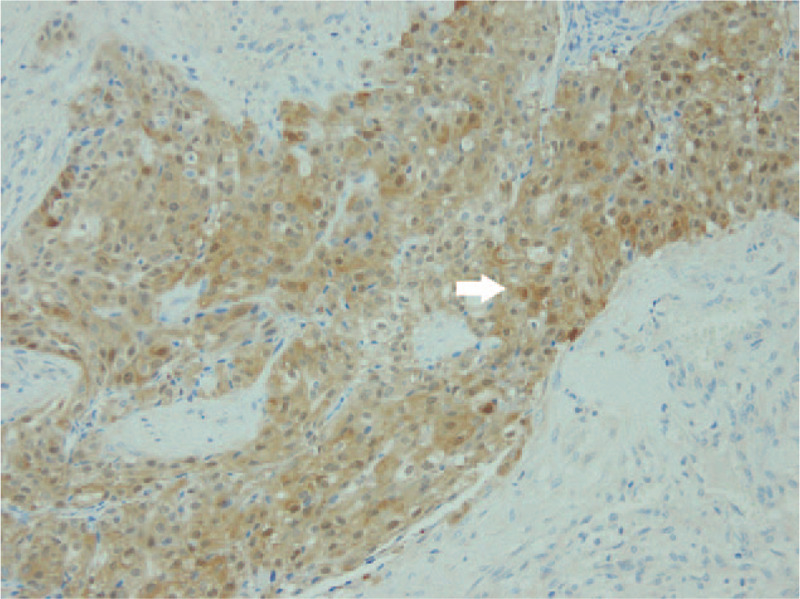
Protein (×200). Many tumor cells are positive for S-100 protein (the white arrow).

A 40-year-old male patient without any specific past history came to the Department of Otolayngology-Head and neck surgery with complains of right infra-auricular mass. Physical examination revealed 4 cm-sized hard, fixed, right infra-auricular mass without tenderness (Fig. 1). To evaluate the character of infra-auricular mass, neck computed tomography (CT) scan was performed. In CT scan, about 3.9 cm-sized poorly defined heterogenous enhancing mass at parotid gland was found (Fig. 2A and Fig. 2B). There was no enlarged or enhancing lymph node in both neck. Fine needle aspiration biopsy was done for right parotid mass and pathologic report was ”lymphocytes only”. With these findings, initial impression was benign or low-grade malignant parotid tumor. With this impression, partial parotidectomy including mass excision was performed. In operative field, as shown in CT scan, intraparotid mass was located in deep lobe (Fig. 3) and severe adhesion between mass and facial nerve found. Meticulous dissection was done and mass was detached from facial nerve without any gross injury. We completed partial partotidectomy without any neck dissection. Final pathologic report revealed secretory carcinoma of parotid gland. Microscopic examination showed a poorly differentiated lobulated tumor. The tumor had a multinodular rearrangement with nodules divided by fibrous septa. The nodules showed a microcystic and tubular pattern. The tumor showed stromal invasion, but no perineural and lymphovascular invasion. The neoplastic cells have round nucleus, with predominant eosinophilic cytoplasm (Fig. 4). Homogenous eosinophilic secretions were identified inside microcystic structure. There was no significant number of mitoses or necrotic areas. The immunophenotype was positive for EMA, vimentin, S-100 protein (Fig. 5). There was no expression of p63, CK5/6, C-KIT. The patient was referred to the Department of Radiologic Oncology to get postoperative radiotherapy. After 15 months of the surgery, the patient followed-up outpatient clinic with negative evidence of disease state. Written informed consent was obtained from the patient for publication of this case report and accompanying images.

## 3 Discussion

MASC was first reported in 2010 by Skálová et al as salivary gland tumor with immunohistochemical finding similar to secretory carcinoma of the breast.^[1]^ Most common similarities between MASC and secretory carcinoma of the breast is the presence of the translocation t(12;15)(p13;q25), which results in the formation of the oncogenic fusion gene ETV6-NTK3,^[3,6]^ that is identifiable by Fluorescence In Situ Hybridization, and also present in other tumors such as infantile fibrosarcoma,^[2,3]^ myelogenous leukemia,^[2]^ and congenital mesoblastic nephroma.^[2,3,7]^ The fusion of the transcriptional regulator gene ETV6 and the membrane receptor kinase-type NTRK3 results in a chimeric tyrosine kinase that activates cell proliferation and increases survival of the tumor cells playing a fundamental role in oncogenesis.^[2,8]^ MASC and secretory carcinoma of the breast also have common immunohistochemical findings such as being positive for S100 protein, EMA, and vimentin, while being negative for 3 receptors (ER/PR/Her2 negative).^[3]^ The most important differential diagnosis for MASC in salivary gland tumor is the acinic cell carcinoma (ACC).^[2]^ ACC is characterized by the presence of large, serous, acinar cells with cytoplasmic Periodic Acid Schiff positive zymogen-like granules that are absent in MASC.^[1]^ MASC is histologically characterized by the proliferation of uniform eosinophilic cells with a vacuolated cytoplasm, growing within a microcystic, macrocystic, and papillary architecture.^[9–11]^ Even though both MASC and ACC are low-grade tumor, MASC is more likely to metastasize to the regional lymph nodes, it should be considered as a more aggressive tumor compared with the regular low grade ACC.^[12,13]^ MASC has a slight preference for male patients, while ACC mainly affects women.^[5]^ Immunophenotypic features that can be used to differentiate MASC from ACC are the expression of protein S100 and positive mammaglobin staining.^[2]^ S100 is strongly positive in MASC, while it is negative in ACC.^[14]^ As for presenting symptoms, MASC usually presents as a painless, nontender mass that increases in size overtime.^[1,15]^ The majority of MASC arise from the parotid gland, accounting for two thirds of the reported cases.^[6]^ MASC is considered a low-grade carcinoma with a favorable prognosis. However, according to Skálová et al, MACS has a moderate risk for local recurrence (15%), lymph node metastases (20%), and a low risk for distant metastases (5%).^[1,4]^ There have been reports of high-grade transformation in MASC in which it becomes a far more aggressive tumor with an accelerated clinical course that results in cancer dissemination and death.^[4]^ The definite diagnosis of MASC could be done by confirming the translocation t(12;15) (p13;q25), which results in the ETV6-NTRK3 gene fusion.^[11]^ However, a negative test for ETV6-NTRK3 gene fusion does not rule out the diagnosis of MASC.^[1,15]^ In those cases, it can also be done with the presence of positive immunohistochemical studies for STAT5, mammoglobin, and S-100 protein.^[14,15]^ The standard treatment modality of MASC is not well defined because the number of reported cases are too small and most studies in the literature are retrospective in nature. The reported disease-free period for MASC ranges from 71 to 115 months, shorter than the one reported in ACC, which is 92–148 months.^[12]^ The treatment of choice in low-grade MACS is surgical resection with or without postoperative radiation therapy. The treatment in high-grade transformation MASC should include radical surgery with neck dissection in addition to adjuvant radiotherapy.^[4]^

Since only about 100 cases of MASC have been reported, further studies and accumulations of data are needed about the behavior, prognostic significance, appropriate diagnostic, and treatment guide. We report the rare case of MASC on parotid gland with clinical features, imaging characteristics, and histopathologic results.

The limitations of this case are lack of genetic studies. The definite diagnosis of MASC is done by confirming the translocation t(12;15) (p13;q25) that was impossible on our institution. Our diagnosis depends mainly on immunohistochemistry findings. Patient follow-up period is relative shorter than disease-free period. Further evaluation after long-term follow up is needed.

## Author contributions

**Formal analysis:** Min Eui Hong.

**Funding acquisition:** Min Eui Hong.

**Investigation:** Min Eui Hong.

**Methodology:** Min Eui Hong, Dong Jin Lee.

**Resources:** Dong Jin Lee.

**Supervision:** Seong Hwan Kim, Dong Jin Lee.

**Validation:** Dong Jin Lee.

**Writing – Original Draft:** Seong Hwan Kim.

**Writing – Review & Editing:** Seong Hwan Kim.
